# Group differences in health literacy are ameliorated in ehealth literacy

**DOI:** 10.1080/21642850.2021.1926256

**Published:** 2021-05-15

**Authors:** Efrat Neter, Esther Brainin, Orna Baron-Epel

**Affiliations:** a Department of Behavioral Sciences, Ruppin Academic Center, Emeq Hefer, Israel; b Faculty of Social Welfare and Health, School of Public Health, University of Haifa, Haifa, Israel

**Keywords:** eHealth literacy, ethnic differences, health literacy, immigration, digital divide

## Abstract

**Background:**

Heath literacy and eHealth literacy are skills that enable individuals to seek, understand and use information in ways which promote and maintain health. The present study examined group differences (ethnicity, immigration) in both literacies and whether there exists an association between the literacies and potential outcomes/gains in health behaviors, health care utilization, perceived health and perceived outcomes of Internet search.

**Methods:**

Participants included 819 Israeli men and women who responded to a nationally representative random-digital-dial (RDD) telephone survey. Respondents were veteran Jews, immigrants from the Former Soviet Union, and Palestinian Citizens of Israel.

**Results:**

Significant differences between the groups were found in health literacy, especially in higher ordered skills, so that the immigrant group was the lowest, after accounting for demographic variables. No significant group differences were found in eHealth literacy. Health literacy was found to be significantly associated with healthcare utilization, perceived health and perceived outcomes of Internet search while eHealth literacy was associated with perceived health and perceived outcomes of Internet search. No interaction was found between group and literacies in the prediction of the outcomes.

**Conclusions:**

Immigration hampers health literacy but differences are ameliorated in eHealth literacy. Finding on association between literacies and outcomes replicated previous ones and the absence of moderation by group attests to the robustness of the models on health literacies.

The present study examined the group (immigration, ethnicity) differences in health literacy and eHealth literacy and whether group membership moderates the association between literacies and varied outcomes in the health domain: healthcare utilizations, health behaviors and perceptions of health and benefits from using the Internet.

## Health literacy, eHealth literacy and their association with health outcomes

Health literacy is defined by the World Health Organization as ‘the cognitive and social skills which determine the motivation and ability of individuals to gain access to, understand and use information in ways which promote and maintain good health’ (World Health Organization, 1998). A definition by the Institute of Medicine focuses on similar capacities that serve making ‘appropriate health decisions’ (Cutilli, 2007; Parker, Ratzan, & Lurie, 2003). This concept was further elaborated as being comprised of three types (Nutbeam, 2000, 2008). The first, functional literacy, involves reading, writing, and basic communication skills that allow functioning effectively in everyday situations. Critical literacy is the ability to critically analyze information and use information to exert greater control over life events and situations. Lastly, interactive literacy is the ability to extract information and derive meaning from different forms of communication and to apply new information to changing circumstances. Rudd, Kirsch, & Yamamato (2004) explicate health tasks that depend on health literacy: activities related to health promotion (e.g. purchase food), health protection (e.g. decide among product options and use products), disease prevention (undergo screening or diagnostic tests), health care and maintenance (e.g. calculate timing for medicine), and system navigation (e.g. locate facilities or apply for benefits).

Historically, the attending physician was the primary source supplying medical and medication-related information, but nowadays a wider range of information sources is available to the public. These information sources include traditional media and electronic media, more specifically, the Internet (Hesse et al., 2005), and the literacy skill necessary to make use of these resources is labeled eHealth literacy. eHealth is the application of information communication technologies across all range of functions involved in the practice and delivery of health care (Ahern, Kreslake, & Phalen, 2006; Eysenbach, 2001). eHealth literacy encompasses basic literacy as well as information, media, health, computer and scientific literacies (the lily model, Norman & Skinner, 2006).

Patients’ health literacy is recognized as crucial in communicating with health care providers and in patient health outcomes (Baker et al., 2007; DeWalt, Dilling, Rosenthal, & Pignone, 2007; Schillinger, 2002; Yin, Dreyer, Foltin, van Schaick, & Mendelsohn, 2007; Zamora & Clingerman, 2011). Poor health literacy is associated with adverse health outcomes: navigation difficulties within the health system, inaccurate or incomplete reports related to medical history, missed doctor appointments, inaccurate use of medications in terms of timing or dosage decreased rates of adherence to chronic illness regimens and increased risk of hospitalization (Baker et al., 1996, 1998, 2002; Williams, Baker, Honig, Lee, & Nowlan, 1998). Health literacy was also found to be associated with functioning in the digital domain, so that low health literacy (and related skills) are negatively related to the ability to understand (Woods & Sullivan, 2019; Zikmund-Fisher, Exe, & Witteman, 2014), evaluate online health information and trust in online health information (Diviani, van den Putte, Giani, & van Weert, 2015). Reviews (Berkman, Sheridan, Donahue, Halpern, & Crotty, 2011; Neter & Brainin, 2019) indicate consistent evidence on the association between health literacy and mortality, persisting after controlling for socioeconomic status, age, and race. The evidence for association with health perceptions (most often self-rated health) or physical functioning, emotional states, quality of life and health behaviors was judged as low or insufficient, mostly due to the cross-sectional design of the studies. Longitudinal findings indicate that much of the association between health literacy and health outcomes are accounted for by cognitive ability (both in old age and during childhood), educational and occupational levels (Mõttus et al., 2014).

Health literacy is measured in various ways. Some tools comprehensively assess tasks in various health domains (health promotion, protection, maintenance, disease prevention, system navigation) (e,g, Health Activity Literacy Scale (HALS; Rudd et al., 2004) while other serve as a screening tool such as S-TOFHLA; Parker, Baker, Williams, & Nurss, 1995), Rapid Estimate of Adult Literacy in Medicine (REALM; Davis et al., 1993), and Newest Vital Sign (NVS; Weiss et al., 2005). Some tools assess performance whereas others are self-reported; the use of the latter increased with the need to administer assessments remotely online (Mackert et al., 2014). Self-report measures that relate both to the above health domains and also to the cognitive skills involved – seeking, understanding (basic literacy and numeracy), evaluating, and applying health information – also exist (e.g. Sørensen et al., 2013; European Health Literacy Scale). This tool was also adapted to other regions (Duong et al., 2017), including Israel (Levin-Zamir, Baron-Epel, Cohen, & Elhayany, 2016), with shorter forms developed recently (Duong et al., 2019).

Having the composite skills of eHealth literacy allows health consumers not only to increase the availability of health information (Knapp, Madden, Wang, Sloyer, & Shenkman, 2011; Mackert et al., 2014; Maroney et al., 2020), translated into knowledge (Stellefson et al., 2019), but also to achieve positive health processes and outcomes, such as quality of life and self-care behaviors (Guo, Hsing, Lin, & Lee, 2021; Kim, Kim, & Choi, 2018; Neter & Brainin, 2012; Stellefson et al., 2019). eHealth literacy has the potential to both protect consumers from harm and empower them to fully participate in knowledge-based decision-making (Norman & Skinner, 2006), but it may also re-enacts the divisions reported on health literacy in the digital domain. Utilizing the Norman and Skinner (2006) scale for measuring self-perceived eHealth literacy (eHEALS) in a representative Israeli sample, Neter and Brainin (2012) found age and education differences between people with high and low eHealth literacy levels, so that the more literate were younger and more educated. Similar findings regarding demographic variables were found in other samples (Choi & Dinitto, 2013; Hoogland et al., 2020; Knapp et al., 2011; Shiferaw, Tilahun, Endehabtu, Gullslett, & Mengiste, 2020). Moreover, differences between high and low eHealth literates were found in information consumption practices and search outcomes, where search outcomes included contact with the attending physician, enhanced use of medical insurance, health behaviors, self-management of health needs, and understanding of the disease/condition (Neter & Brainin, 2012). eHealth literacy was thus concluded to be a ‘second-level’ digital divide, constituting a capital-enhancing gap where access equity was reached and powering the ‘third level’ digital divide of achieving tangible outcomes from Internet use (Van Deursen & Helsper, 2015). Tangible outcomes could be adoption of health behaviors (Guo et al., 2021; Kim et al., 2018; e.g. Mitsutake, Shibata, Ishii, & Oka, 2016), healthcare utilization (Maroney et al., 2020) and ultimately health (or perceived health, see Shiferaw et al., 2020) though a recent review concluded that there are currently too few studies attesting on these associations (Neter & Brainin, 2019). eHealth literacy has been measured thus far mostly with eHEALS (Griebel et al., 2017; Karnoe & Kayser, 2015; Neter & Brainin, 2019), though its shortfalls chiefly in assessing ‘participative Internet’ (i.e. Web 2.0) as opposed to ‘passive Internet’ (i.e. Web 1.0) have been noted (Griebel et al., 2017; Norman, 2011; Van der Vaart & Drossaert, 2017). Though several new measures were published (e.g. Chew, Cheng, Grant, & Bastidas, 2014; Guo et al., 2021; Jones, 2013; Koopman, Petroski, Canfield, Stuppy, & Mehr, 2014; Van der Vaart & Drossaert, 2017), they were mostly not used or reworked by other researchers (Griebel et al., 2017; Karnoe & Kayser, 2015). A new initiative for incorporating eHealth literacy into the measurement of health literacy (Van Den Broucke et al., 2020) may both update the assessment of eHealth literacy to include participative activities and yield wider uptake of the tool by the many researchers involved in the initiative.

## Group differences in health and eHealth literacies

Previous work has documented differences in both health literacy and eHealth literacy among groups, varying in ethnicity, social economic status (operationalized as education or income) age or health status (Choi & Dinitto, 2013; Knapp et al., 2011; Tennant et al., 2015; Wångdahl, Lytsy, Mårtensson, & Westerling, 2015). The current work seeks to examine group differences on an Israeli multicultural sample, which allows looking into differences between veteran Jews, immigrants from the former Soviet Union (FSU), and Palestinian Citizens of Israel (PCI).

Immigration is a life event that places individuals at a relative disadvantage (e.g. Amit & Chachashvili-Bolotin, 2018) that may be more associated with health literacy than eHealth literacy. Health literacy, as envisioned by Nutbeam (2000), involves communication, critical and interactive skills in which immigrants can be at a relative disadvantage, primarily due to language deficiency. Indeed, Levin-Zamir & Baron-Epel (2016) have documented differences in health literacy between veteran Israelis and FSU immigrants so that the latter possess lower health literacy. Conversely, eHealth literacy occurs in a virtual domain that may ameliorate these deficiencies, especially if some of the information is sought and appraised in a language of choice rather than in the language of the host country. Indeed, focusing on capital-enhancing forms of Internet use, no differences in human capital enhancement (seeking information on news, banks, people) were recorded between veteran Jews and FSU immigrants and an advantage to immigrants was documented in social capital enhancement Internet uses (using chatrooms, forums, Messenger, Skype), even before controlling for socio-demographic attributes and Hebrew language proficiency (Lissitsa & Chachashvili-Bolotin, 2014). Consequently, group differences between veteran Israelis and FSU immigrants are expected in health literacy but not in eHeath literacy.

The second group of interest is a disadvantaged minority of Palestinians in Israel. This group is distinctive culturally, having a different language (Arabic), affiliating mostly with the Muslim religion, and having autonomous institutions in various domains such as mass media, religion and education (Smooha, 1997). Thought PCIs are full citizens of Israel, they are disadvantaged in education and other social-economic indicators, relative to veteran Jews, and are frequently discriminated against (Baron-Epel, Berardi, Bellettiere, & Shalata, 2017; Haberfeld & Cohen, 2007; Lissitsa & Chachashvili-Bolotin, 2019). Due to lower educational attainment and previous findings on health literacy among Israeli groups (Levin-Zamir et al., 2016), PCI are expected to have lower health literacy.

Previous findings on Internet access and internet use among PCI are mixed and evolving. Internet access, also labeled the ‘first digital divide’, was found to be still lagging among PCI, compared to the majority group (Avidar, 2009; Ganayem, Rafaeli, & Azaiza, 2009; Lev-On & Lissitsa, 2010; Lissitsa & Chachashvili-Bolotin, 2014; Mesch & Talmud, 2011), supporting the stratification hypothesis that information and communication technologies adoption replicates existing social inequalities. Still, a more recent study reported that the disadvantage disappeared after controlling for socio-demographic variables (Lissitsa, 2015; Lissitsa & Chachashvili-Bolotin, 2014). Second-level digital gaps focus on digital skills and use/activities; differences in these activities, such as capital enhancement and recreational activities, were found to persist between PCI and the majority group, even after controlling for socio-demographic variables (Lissitsa & Chachashvili-Bolotin, 2014). However, Mesch (2012) found PCI more likely to use the Internet to make new contacts and to expand their business contacts, and interpreted his findings to support the diversification hypothesis positing that computer-mediated communication provides a platform for minorities in overcoming social constraints. In the health domain, though PCI are most often serviced by local clinics providing primary care and operating primarily in Arabic, accessing some specialist may sometimes involve travel to larger Jewish cities, thus incurring language barriers and transportation costs. These challenges may also explain why PCI are more likely to access health information online than the majority group: such information is accessible in Arabic and it can potentially eliminate transportation costs (Mesch, 2015). Thus, compared with the majority group, PCI were expected to have lower health literacy and higher eHealth literacy.

## The present study

The present study aims to examine the group differences in health literacy and eHealth literacy and whether group membership moderates the association between literacies and outcomes.

First, the health literacy and eHealth literacy of FSU immigrants and PCI was compared to those of veteran Jews. We hypothesized lower scores in health literacy for PCI (H1) and FSU (H2), compared to veteran Jews, and higher scores in eHealth literacy for PCI (H3), compared to veteran Jews.

Second, a moderation of group membership in the association between literacies and outcomes was examined. Outcomes examined were perceived outcomes of information use, perceived health, health care utilization and health behaviors, all constituting potential ‘third level divide’, defined as accrued outcomes or benefits from digital use. Though associations between the literacies and health outcomes were recorded in this sample and in the literature (e.g. Guo et al., 2021; Hoogland et al., 2020; Kim et al., 2018; Levin-Zamir et al., 2016; Neter, Brainin, & Baron-Epel, 2018; Neter & Brainin, 2019; Shiferaw et al., 2020), no findings on an interaction between literacies and group membership on health outcomes were previously reported; hence, the present investigation is explorative on the matter.

## Methods

### Data collection and sample characteristics

Data analyzed in this paper was collected from a nationally representative random-digital-dial (RDD) telephone survey of the Israeli adult population (21 and older) conducted in November 2014 (landlines and mobile combined).

Calls were placed to 1789 residential households or mobile phones to identify 1628 eligible potential respondents, of whom 819 agreed to be interviewed, representing 50.3% response rate. The interviews were conducted by professional interviewers, speakers of Hebrew, Arabic or Russian, who went through a special training session to familiarize them with the questionnaire's terminology. The interviewers conducted the telephone survey using Computer Assisted Telephone Interviewing software.

A total of 683 participants were Jews, 79 among them FSU immigrants since the 1990s, and 136 were PCI.

### Measurements

*eHealth Literacy* was assessed by the eHEALS (Norman & Skinner, 2006). The scale comprises of eight items on a 5-point Likert scale (1 = strongly disagree,to 5 = strongly agree). The scale was previously translated to Hebrew, Arabic and Russian (Neter & Brainin, 2012). A composite score was created by averaging the means of the two subscales of seeking and appraising, found as dimensions in this sample (Neter, Brainin, & Baron-Epel, 2015). The scale was assessed only among respondents who reported using the Internet for health purposes (*n*=403). The Cronbach internal reliability of the overall scale was α=0.89 while reliabilities for both subscales were α = 0.83.

*Health Literacy* was assessed by the European Health literacy Scale (HLS-EU) (Sørensen et al., 2013). The 15-items short version of the scale was used. The scale was translated to Hebrew, Russian and Arabic and validated by Levin-Zamir et al. (2016), using a 16-items questionnaire, and one item was deleted in the present administration due to comprehension problems of respondents. The 15 items are mostly on a 5-point Likert scale (1 = strongly disagree to 5 = strongly agree). A composite score was created by averaging the means of the three subscales of seeking, understanding and appraising/applying, found as dimensions in this sample (Neter et al., 2015). The Cronbach internal reliability of the overall scale was α=0.86, and reliabilities for the subscales were α = 0.72 (seeking), α = 0.85 (understanding), and α = 0.83 (appraising/applying).

*Perceived outcomes* of seeking health information on the Internet were examined by asking ‘Do you agree or disagree that seeking health information on the Internet … ?’ A list of 9 outcomes, adapted from (Baker, Wagner, Singer, & Bundorf, 2003). It was translated into all the three languages and used previously in an Israeli sample (Neter & Brainin, 2012). Items included: improved your ability to manage your health needs; enabled you to ask your physician questions resulting from the information you acquired on the Internet; enabled you to show your physician the information that you retrieved; raised your sense of power in your encounter with the physician; improved your understanding of the symptoms, conditions, or treatments in which you were interested; updated your knowledge in health innovations; led you to take independent steps (such as seeing a specialist, or changing an exercise regimen or eating habits); enabled you to think about alternative treatment options; and made you more aware of patients’ insurance rights. A 5-point response scale was used (from strongly agree to disagree). The total mean score for each participant’s outcome perception was computed with a higher score representing a more positive perception of the health benefits derived from Internet use (α = .87).

*Health behaviors* (Levin-Zamir et al., 2016) were tapped by asking respondents about smoking and physical activity. The smoking item ranged from ‘never smoked’ (=1) to ‘smokes daily’ (=4). The physical activity item tapped frequency of engaging in a 30-minutes activity such as walking, running, swimming, or other athletic activity. The item was on a four-point scale ranging from ‘daily’ to ‘not at all’ (a fifth possible option ‘cannot exercise’ was not selected by any respondent).

*Use of healthcare services* was measured by asking respondents whether they have visited their general practitioner, visited a specialist, have attended an emergency room and were hospitalized in the past year. Responses were provided on a three-point response scale, including ‘no’, ‘yes, once or twice’, and ‘yes, three times or more’ (ICDC, 2015; Levin-Zamir et al., 2016). For each respondent, a total mean score was computed. The internal reliability of the scale was α = 0.66.

*Perceived health*, or self-rated health (SRH) is a widely used health measure (Idler & Benyamini, 1997) in which respondents are asked to evaluate their own health condition compared with other people their age and gender. The response options to this single item ranged from poor (=1) to very good (=5).

Socio-demographic information on ethnicity, immigration status, year of immigration, age, gender and attained education was obtained as part of the background variables. Education was measured by eight categories ranging from elementary school to graduate education using Israeli Bureau of Statistics scale.

### Data analysis

We first performed univariate descriptive analyses, characterizing participants. Then, we compared the health and eHealth literacies across the population groups. In examining the possible moderation of the association between literacies and health process by group membership, we initially computed Pearson correlations between the literacies and the outcome variables, and proceeded to multivariate analyses in the outcome variables with significant bivariate associations with the literacies. In multivariate hierarchical regressions, we examined the moderation of group in the association between literacies and outcomes: the first step comprised of demographic characteristics (age, gender, education, and group). In the second step we added the literacies, and the third step added the interaction terms between group and literacies (health and eHealth). In the outcome variable of healthcare utilization, self-rated health, as a good approximation of health status (Idler & Benyamini, 1997), was also included in the first step. Data analysis was carried out using SPSS v. 23.0 (IBM Corp., 2015).

### Ethics Statement

This was a public opinion survey, which at the time (2014) did not require an IRB.

## Results

### Descriptive sample statistics

Table 1 reports background variables across and by group. Overall, respondents included slightly more women than men and more so in the FSU group. Respondents in the FSU group were older, attained more formal education and felt less healthy than veteran Jews and PCI.

**Table 1.** Description of Respondents (*N*=819) on Demographic Characteristics.

	Total	Veteran Jews	Former FSU	PCI	χ^2^ / F *P* value	
	*N*=819	*N*=604	*N*=79	*N*=136		
Variable						
Age, in years (mean ± sd)	49.72 ± 17.00	50.39 ± 16.74	56.79 ± 19.68	42.5 ± 13.62	19.29	0.000
Gender, women, *N* (%)	424 (51.8)	304 (50.3)	51 (64.6)	69 (50.7)	5.73	0.57
Self-rated health, mean (SD)	3.97 (.98)	4.02 (.96)	3.62 (.87)	3.96(1.13)	5.47	0.004
Education					17.51	0.000
Elementary to full secondary, N (%)	368 (45.0)	284 (47.1)	18 (22.8)	66 (48.5)		
Post-secondary, N (%)	450 (55.0)	319 (52.9)	61 (77.2)	70 (51.5)		
Internet Use (Yes), N (%)	699 (73.3)	452 (74.8)	57 (72.2)	91 (66.9)	3.61	0.164

### Group differences in health literacy and eHealth literacy

**Figure 1.**
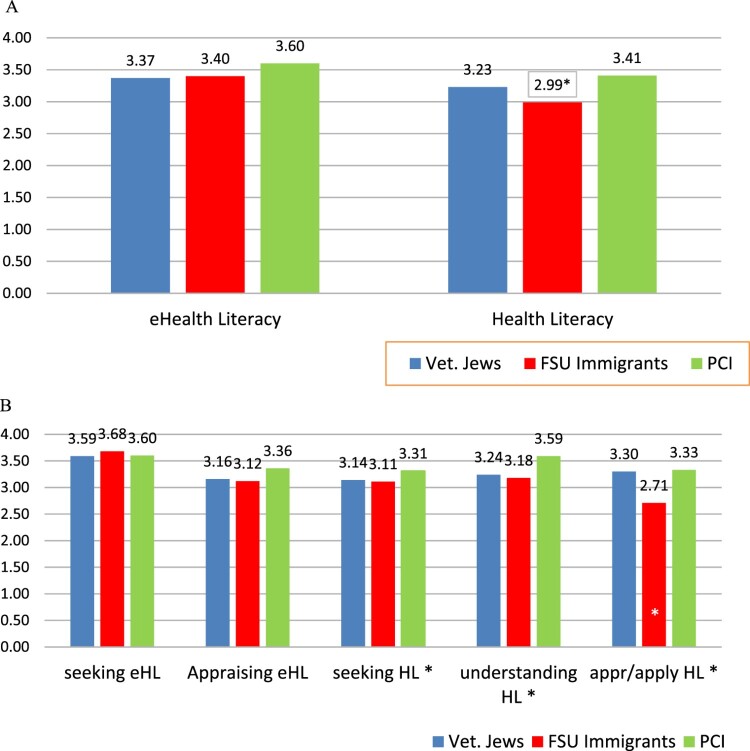
(A) Group differences in the indices of health literacy and eHealth literacy (*n*=627). (B) Group differences in the dimensions of health literacy and eHealth literacy (*n*=342).

The three groups – veteran Jews, PCI and FSU immigrants – were compared on their perceived health literacy and eHealth literacy. Figure 1 presents mean groups in the literacies and their dimensions, where panel A includes the indices and panel B contains the dimensions. The figure indicates that the immigrant group scored lower in health literacy, both in the index and in the dimensions, with the biggest difference emerging in the appraise/apply factor.

A significant difference was found in health literacy among the groups, *F*(2, 813) = 21.21, *p*=.000, η^2 ^= .050. The significant difference in health literacy between the groups remained after adjusting for age, gender, and education (β = 0.82, *SE* = 0.22, 95% CI [0.008–0.095], *p* = 0.021). The planned contrast comparing FSU (M = 2.99, SD = .50) to veteran Jews (M = 3.23, SD = .46) was significant: *t*(96.12) = 4.20, *p* < 0.001, thus supporting H2. Moreover, the significant difference emerged in all the sub-scales – seeking, understanding, and appraising/applying: *F*(2, 768) = 3.78, *p *= .023, η^2 ^= .010; *F*(2, 804) = 17.13, *p* < .001, η^2 ^= .041 and *F*(2, 813) = 44.30, *p* <.001, η^2 ^= .098, respectively. The planned contrast comparing PCI (M = 3.41, SD = .50) to veteran Jews (M = 3.23, SD = .46) on health literacy was not conducted as the direction of the difference was contrary to the hypothesized direction, thus not supporting H1.

No significant differences were found among the groups in eHealth literacy, *F*(2,395) = 1.93, *p *= .146, η^2 ^= .010, yet the planned contrast comparing PCI (M = 3.60, SD = .72) to veteran Jews (M = 3.37, SD = .82) on eHealth literacy was significant, *t*(85.92) = 2.14, *p* < 0.035, thus supporting H3. No significant differences between the groups emerged in the seek and appraise dimensions comprising eHealth literacy, *F*(2,395) = 2.01, *p *= .136, η^2 ^= .010 and *F*(2,395) = 1.43, *p *= .241, η^2 ^= .007, respectively.

### Association between literacies and health-related processes and its moderation by group

In order to examine whether group moderates the associations between health literacy and eHealth literacy (both perceived) and health-related processes, we first computed Pearson correlations between health behaviors (smoking and physical activity), health care utilization, perceived outcomes of information search and perceived health. The results are displayed in Table 2. As can be seen, health literacy was significantly associated with all variables except smoking. eHealth literacy is significantly related to self-rated health (*r=*0.13, *p=*0.01) and to outcomes gained from the Internet search (*r=*0.40, *p=*0.000), but not to health behaviors and to healthcare utilization (*p’s>.*05).

**Table 2.** Intercorrelations between main variables.

1. Health literacy	–							3.23	.47
2. eHealth Literacy	.39***	–						3.41	.80
3. Physical Activity	.08*	-.02	–					2.91	1.29
4. Smoking	.07	.05	.08*	–				0.82	1.13
5. Health care Utilization	-.07*	-.01	.01	.03	–			1.64	.39
6. Internet search outcomes	.12*	.40***	.08	.09	.09	–		3.04	.89
7. Self-rated health	.33***	.13**	.17***	.05	.28***	0.4	–	4.17	.89

*p < .05; **p < .01; ***p < .001.

Further analyses were carried out on the outcome variables found to be associated with literacies in the above bivariate analysis (Table 3). In order to test the group moderation of the association between literacies and self-rated health, a multivariate linear regression was conducted. Background variables were included in the first step (age, gender, education and group) and health literacy and eHealth literacy were included in the second step. The first step, comprising of background variables was significantly associated with perceived health, *F*(4,362) = 7.23, *p* < 0.001, explaining 6.4% of the variance, and in this step age and gender were significantly associated with perceived health, so that older people (*β *= –.23, *t *= 4.49, *p* < 0.001) and women (*β*=–.13, *t*=2.63, *p*=0.009) perceived their health as less good. Group was not significantly associated with self-rated health (*β*=–.02, *t*=−0.47, *p*=0.640). The second step, adding the literacies, was statistically significantly associated with perceived health: it explained 14.8% of the variance, *F*(6,360) = 11.56, *p*=0.000. Age and gender remained significant associated variables and health literacy (*β*=.31, *t*=5.90, *p *< 0.001) also was significantly associated with self-rated health. Group was not significantly associated with perceived health (*β *= -.05, *t *= −1.06, *p *= .292). The third and last step added negligibly (0.7%) to the explained variance and the interaction between group and each of the literacies was not significant.

**Table 3.** Hierarchical Linear Regression on Self-rated Health, Healthcare Utilization and Perceived Outcomes of Internet Use; Testing Moderation by Group.

	Step 1			Step 2			Step 3		
	B	SE B	β	B	SE B	β	B	SE B	β
*Self-rated health (n=316)*									
Gender	−.22	.08	−.13**	−.18	.08	−.11*	−.18	.08	−.11*
Age	−.01	.01	−.23***	−.01	.01	−.25***	−.01	.01	−.25***
Education	.03	.02	.06	.02	.02	.04	.02	.02	.03
Group	−.03	.06	−.02	−.06	.06	−.05	−.10	.06	−.09
Health Literacy				.63	.11	.31***	.29	.23	.14
eHealth Literacy				.−04	.05	−.04	.02	.12	.02
Health literacy * group							.11	.07	.20
eHealth literacy * group							−.03	.06	−.06
									
Adjusted *R*^2^	0.064			0.148			0.150		
*R*^2^ change				.088			.007		
Healthcare utilization (*n* = 365)									
Gender	.12	.04	.16**	.12	.04	.16**	.12	.04	.16**
Age	.00	.00	.04	.00	.00	.04	.00	.00	.04
Education	.01	.01	.03	.01	.01	.03	.01	.01	.03
Self-rated health	−.06	.02	.14*	−.06	.03	−.14*	−.06	.03	−.14*
Group	−.02	.03	.−05	−.02	.03	−.05	−.03	.03	−.05
Health Literacy				.01	.05	.01	−.05	.11	−.06
eHealth Literacy				.00	.03	.00	.07	.06	.16
Health literacy * group							.02	.03	.08
eHealth literacy * group							.−04	.03	−.17
									
Adjusted *R*^2^	0.056			0.056			0.061		
*R*^2^ change				.000			.005		
*Perceived Outcomes of Internet Use (n=369)*									
Gender	.05	.09	.03	.02	.09	.11	.02	.09	.01
Age	−.01	.00	−.08	−.00	.00	−.00	.−00	.00	−.04
Education	.03	.03	.06	−.11	.02	−.02	−.01	.03	−.03
Group	.11	.07	.09	.09	.06	.07	.09	.07	.07
Health Literacy				−.09	.12	−.04	−.20	.25	−.09
eHealth Literacy				.47	.06	.41***	.65	.13	.57***
Health literacy * group							.04	.07	.06
eHealth literacy * group							−.11	.07	−.18
Adjusted R^2^	0.020			0.171			0.177		
R^2^ change				.151			.006		

Note: ***p<.05; ***p<.01; ***p<.001; *R*^2^ change refers to added explained variance between the steps.

A similar analysis was carried out for healthcare utilization. Background variables were included in the first step (age, gender, education, self-rated health and group) and health literacy and eHealth literacy were included in the second step. Only the first step significantly predicted health care utilization, *F*(5,359) = 4.22, *p < *0.001, explaining 5.6% of the variance, and in this step self-rated health and gender significantly predicted perceived health, so that people who perceive their health as less than others’ (*β *= .14, *t*=2.55, *p *= 0.011) and women (*β *= .16, *t *= 3.03, *p *= 0.003) used the healthcare system to a larger extent. The addition of the literacies in the second step did not add to the explanation of the variance of healthcare utilization (δ *r*^2^=.000, *F*(2, 357) = 0.018, *p* = .983). The third and last step added negligibly (0.5%) to the explained variance: the interaction between group and each of the literacies was not significant.

A third multivariate linear regression was conducted on perceived outcomes gained from Internet search. Background variables were included in the first step (age, gender, education and group) and health literacy and eHealth literacy were included in the second step. The interactions between the literacies and the group were added in the third step. The first step, comprising of background variables, did not significantly predict perceived outcomes, *F*(4,364) = 1.90, *p *= 0.109, explaining a mere 2.0% of the variance, and in this step none of the variables significantly predicted the outcome. The second step, comprising of the literacies, was statistically significant in predicting the perceived outcomes gained from information search on the Internet: it explained 17.1% of the variance, *F*(6,362) = 12.46, *p < *0.001. The only significantly predicting variable in this step was eHealth literacy (*β*=.57, *t*=7.74, *p <*0 .001). A moderation analysis indicated that the interaction between eHealth literacy and group membership was nonsignificant (*β *= –.18, *t *= −1.56, *p *= 0.119), so that the association between eHealth literacy in all three groups was similar. A final multivariate linear regression was conducted on physical activity. None of steps added to explained variance significantly (*p*>.05).

## Discussion

The study examined population group differences in health literacy and eHealth literacy and then the potential moderation of the association between literacies and health outcomes by group. The study documented group differences in health literacy in an Israeli representative sample, so that immigrants (compared to non-immigrants) had lower health literacy (H2). No group (ethnicity, immigration) differences were recorded in eHealth literacy (H3). Though associations between literacies and health-related outcomes were found, these associations were not moderated by group.

The results on differences between immigrants and veterans in health literacy echo previous findings, recorded in several countries, including Israel (Levin-Zamir et al., 2016), the location of the present sample. It is of interest that the group differences documented were between the immigrant group and the majority group, even though health literacy is associated with education (Paasche-Orlow & Wolf, 2007) and the FSU immigrant group had attained more education than the majority group. Apparently, immigration impedes health literacy, even in the case of a highly educated group. This could be due to health literacy being anchored in a cultural and language context. This was reflected in the scale used (European Health literacy Scale (HLS-EU)) which included items on consuming mass media and other information likely to be conveyed mostly in the language of the majority group. The high health literacy of PCI, compared to veteran Jews, was contrary to previous findings (Levin-Zamir et al., 2016) and to H1, and thus could not be tested in a direct contrast. This finding has several explanations. It could be due to the younger age of PCI, compared to the other groups in the sample, as well as to the relatively high educational attainment of PCI in this sample, compared with the national average (Israel Central Bureau of Statistics, 2020b). Another potential explanation is the access to many health services and messages in their native language (i.e. Arabic) such as primary care clinics and Arabic-speaking media, facilitating health literacy. Lastly, the higher reported health literacy could be attributed to acquiescence bias in responding to survey questions, documented to be higher among Arabic speakers in Israel (Baron-Epel, Kaplan, Weinstein, & Green, 2010).

Conversely, the absence of difference in eHealth literacy among the groups, attests to the empowering and capital-enhancing qualities of the Internet (Lissitsa & Chachashvili-Bolotin, 2014), especially when compared to the difference in health literacy. Language and navigation challenges in a health system waned in the digital arena, which at the time of the study provided mainly information on services and health conditions and did not yet require active participation. Under these circumstances, language was chosen and not determined. This is the novel finding of the present study. It reverberates findings on digital literacy and its association with language literacy (Lissitsa & Chachashvili-Bolotin, 2014). It is unclear whether this finding will persist when afforded services require a two-way interaction in designated languages and whether health providers will afford services in several languages. In Israel, only basic services are currently offered in five languages (Hebrew, Arabic, Russian, English, and French). Lastly, gaps in Internet use, specifically lower use among PCI, is still evident today (Israel Central Bureau of Statistics, 2020a), though use has increased among all groups and the gap between the groups decreased (80.7%, 82.8% and 84.9% for Arabs, Israeli veterans, and FSU, respectively). These differences may be due to reduced access in rural/peripheral areas and to differential uptake associated with education (FSU having the highest attainment) and income.

The findings on the positive association between health literacy and health behaviors, health care utilization and self-rated health are mostly consistent with previous studies and with systematic reviews on health literacy (Berkman et al., 2011; Neter & Brainin, 2019). Likewise are the findings on eHealth literacy, a research domain with fewer studies (Neter & Brainin, 2019), where the highest positive association found was with perceived benefits of Internet use. It is possible that benefits from eHealth literacy are in fewer domains than is the case in health literacy, yet more evidence is needed for such interpretation.

The absence of group moderation to the association between the health literacies and health processes (i.e. healthcare utilization, self-rated health, perceived benefits of Internet use) may indicate that the direction and strength of the associations are solid. Though the ethnic and immigration health divisions in Israel are substantial (Daoud, Soskolne, Mindell, Roth, & Manor, 2018), the associations were similar among the three groups for varied health processes, demonstrating the robustness of models of health literacy (Bailey et al., 2014; Paasche-Orlow & Wolf, 2007) and eHealth literacy (Norgaard et al., 2015; Norman & Skinner, 2006) which theorize association between health literacies, health processes and health outcomes. Lastly, traditional social determinants of health variables, i.e. age and gender, were mostly associated with ‘established’ health outcomes such as self-rated health and healthcare utilization in the first step of the regression analyses, while no demographic variable were associated with perceived outcomes of Internet use, a new and context-specific variable.

### Research strengths, limitations and future directions

This study used a representative sample and examined health literacy and eHealth literacy in a heterogenous society, affording the examination of group differences. It also used sound and prevailing measures to assess the literacies at question. The methodology of examining the potential moderation of an association by a group is also well-established.

Still, the present study is limited, primarily by its design. The cross-sectional survey precludes inferring about causality or even the direction of the association. The reliance on self-reports and a single source of information is also detrimental. Though self-reports are associated with actual performance in both health literacy and eHealth literacy (Karnoe & Kayser, 2015; Neter & Brainin, 2017; Nguyen, Paasche-Orlow, & McCormack, 2017) and are prevalent, other designs and methodologies could contribute a better understanding of health literacy and eHealth literacy.

Inclusion of health literacies measures (performed or perceived) in large longitudinal studies with representative samples (e.g. Survey of Health, Ageing and Retirement in Europe (SHARE) or Midlife in the United States: A National Longitudinal Study of Health and Well-being (MIDUS)) could afford inferring about the direction of the association. Another methodology worth pursuing is machine learning of digital activities. Data mining could measure health-related Internet use and eHealth literacy more accurately as well as associate it with other activities, benefits or health status.

In conclusion, the study documented a significant difference in health literacy between an immigrant group and the veteran majority group. There were no group differences in the context of ethnicity or immigration in eHealth literacy, attesting to the capital-enhancing promise of Internet use. The association between the literacies and health processes varied in magnitude between the different outcomes but was similar across the examined groups.

## References

[CIT0001] Ahern, D. K., Kreslake, J. M., & Phalen, J. M. (2006). What is eHealth (6): Perspectives on the evolution of eHealth research. *Journal of Medical Internet Research*, *8*(1), e4. doi:10.2196/jmir.8.1 16585029 PMC1550694

[CIT0002] Amit, K., & Chachashvili-Bolotin, S. (2018). Satisfied with less? Mismatch between subjective and objective position of immigrants and native-born men and women in the labor market. *Frontiers in Sociology*, *3*, 33. doi:10.3389/fsoc.2018.00033

[CIT0003] Avidar, R. (2009). Social media, societal culture and Israeli public relations practice. *Public Relations Review*, *35*(4), 437–439. doi:10.1016/j.pubrev.2009.06.002

[CIT0004] Bailey, S. C., Brega, A. G., Crutchfield, T. M., Elasy, T., Herr, H., Kaphingst, K., … Schillinger, D. (2014). Update on health literacy and diabetes. *Diabetes Educator*, *40*(5), 581–604. doi:10.1177/0145721714540220 PMC4174500 24947871

[CIT0005] Baker, D. W., Gazmararian, J. A., Williams, M. V., Scott, T., Parker, R. M., Green, D., … Peel, J. (2002). Functional health literacy and the risk of hospital admission among Medicare managed care enrollees. *American Journal of Public Health*, *92*(8), 1278–1283. doi:10.2105/AJPH.92.8.1278 12144984 PMC1447230

[CIT0006] Baker, D. W., Parker, R. M., Williams, M. V., & Clark, W. S. (1998). Health literacy and the risk of hospital admission. *Journal of General Internal Medicine*, *13*(12), 791–798. doi:10.1046/j.1525-1497.1998.00242.x 9844076 PMC1497036

[CIT0007] Baker, D. W., Parker, R. M., Williams, M. V., Pitkin, K., Parikh, N. S., Coates, W., & Imara, M. (1996). The health care experience of patients with low literacy. *Archives of Family Medicine*, *5*(6), 329–334. http://www.ncbi.nlm.nih.gov/pubmed/8640322%5Cnfiles/139/8640322.html 8640322 10.1001/archfami.5.6.329

[CIT0009] Baker, D. W., Wolf, M., Feinglass, J., Thompson, J., Gazmararian, J., & Huang, J. (2007). Health literacy and mortality among elderly persons. *Archives of Internal Medicine*, *167*(14), 1503–1509. doi:10.1001/archinte.167.14.1503 17646604

[CIT0008] Baker, L., Wagner, T. H., Singer, S., & Bundorf, M. K. (2003). Use of the Internet and e-mail for health care information. *JAMA*, *289*(18), 2400–2406. doi:10.1001/jama.289.18.2400 12746364

[CIT0010] Baron-Epel, O., Berardi, V., Bellettiere, J., & Shalata, W. (2017). The relation between discrimination, sense of coherence and health varies according to ethnicity: A study among three distinct populations in Israel. *Journal of Immigrant and Minority Health*, *19*(6), 1386–1396. doi:10.1007/s10903-016-0449-4 27325224

[CIT0011] Baron-Epel, O., Kaplan, G., Weinstein, R., & Green, M. S. (2010). Extreme and acquiescence bias in a bi-ethnic population. *European Journal of Public Health*, *20*(5), 543–548. doi:10.1093/eurpub/ckq052 20439322

[CIT0012] Berkman, N. D., Sheridan, S. L., Donahue, K. E., Halpern, D. J., & Crotty, K. (2011). Low health literacy and health outcomes: An updated systematic review. *Annals of Internal Medicine*, *155*(2), 97–107. doi:10.7326/0003-4819-155-2-201107190-00005 21768583

[CIT0013] Chew, F., Cheng, Z., Grant, W., & Bastidas, C. E. C. (2014). Developing a new scale to measure e-health literacy. *World congress on social media*, mobile apps, and internet/Web 2.0 in health, Medicine and biomedical research.

[CIT0014] Choi, N. G., & Dinitto, D. M. (2013). The digital divide among low-income homebound older adults: Internet use patterns, ehealth literacy, and attitudes toward computer/Internet use. *Journal of Medical Internet Research*, *15*(5), 1–23. doi:10.2196/jmir.2645 PMC3650931 23639979

[CIT0015] Cutilli, C. C. (2007). Health literacy in geriatric patients: An integrative review of the literature. *Orthopaedic Nursing*, *26*(1), 43–48. http://www.ncbi.nlm.nih.gov/pubmed/17273109 17273109 10.1097/00006416-200701000-00014

[CIT0016] Daoud, N., Soskolne, V., Mindell, J. S., Roth, M. A., & Manor, O. (2018). Ethnic inequalities in health between Arabs and Jews in Israel: The relative contribution of individual-level factors and the living environment. *International Journal of Public Health*, *63*(3), 313–323. doi:10.1007/s00038-017-1065-3 29273838

[CIT0017] Davis, T.C, Long, S. W, Jackson, R.H, Mayeaux, E.J, George, R.B, Murphy, P.W, & Crouch, M.A(1993). Rapid estimate of adult literacy in medicine: a shortened screening instrument. Family medicine, *25*(6), 391–395. 8349060

[CIT0018] DeWalt, D. A., Dilling, M. H., Rosenthal, M. S., & Pignone, M. P. (2007). Low parental literacy is associated with worse asthma care measures in children. *Ambulatory Pediatrics*, *7*(1), 25–31. doi:10.1016/j.ambp.2006.10.001 17261479 PMC1797805

[CIT0019] Diviani, N., van den Putte, B., Giani, S., & van Weert, J. C. M. (2015). Low health literacy and evaluation of online health information: A systematic review of the literature. *Journal of Medical Internet Research*, *17*(5), 1–17. doi:10.2196/jmir.4018 PMC4468598 25953147

[CIT0020] Duong, T. V., Aringazina, A., Baisunova, G., Nurjanah, P., Pham, T. V., Truong, K. M., … Chang, P. W. (2017). Measuring health literacy in Asia: Validation of the HLS-EU-Q47 survey tool in six Asian countries. *Journal of Epidemiology*, *27*(2), 80–86. doi:10.1016/j.je.2016.09.005 28142016 PMC5328731

[CIT0021] Duong, T. V., Aringazina, A., Kayupova, G., Nurjanah, T. V., Pham, K. M., Truong, T. Q., … Chang, P. W. S. (2019). Development and validation of a new short-form health Literacy Instrument (HLS-SF12) for the general public in six Asian countries. *Health Literacy Research and Practice*, *3*(2), e91–e102. doi:10.3928/24748307-20190225-01 31294310 PMC6607763

[CIT0022] Eysenbach, G. (2001). What is e-health? *Journal of Medical Internet Research*, *3*(2), e20. doi:10.2196/jmir.3.2.e20 11720962 PMC1761894

[CIT0023] Ganayem, A., Rafaeli, S., & Azaiza, F. (2009). Digital divide: Internet usage within Israeli Arab society. *Megamot*, *46*(1–2), 164–196.

[CIT0024] Griebel, L., Enwald, H., Gilstad, H., Pohl, A. L., Moreland, J., & Sedlmayr, M. (2017). Ehealth literacy research—Quo vadis? *Informatics for Health and Social Care*, 1–16. doi:10.1080/17538157.2017.1364247 29045164

[CIT0025] Guo, S. H. M., Hsing, H. C., Lin, J. L., & Lee, C. C. (2021). Relationships between mobile ehealth literacy, diabetes self-care, and glycemic outcomes in Taiwanese patients with type 2 diabetes: Cross-sectional study. *JMIR MHealth and UHealth*, *9*(2), 1–13. doi:10.2196/18404 PMC7895642 33544088

[CIT0026] Haberfeld, Y., & Cohen, Y. (2007). Gender, ethnic, and national earnings gaps in Israel: The role of rising inequality. *Social Science Research*, *36*(2), 654–672. doi:10.1016/j.ssresearch.2006.02.001

[CIT0027] Hesse, B. W., Nelson, D. E., Kreps, G. L., Croyle, R. T., Arora, N. K., Rimer, B. K., & Viswanath, K. (2005). Trust and sources of health information. *Archives of Internal Medicine*, *165*, 2618–2624. doi:10.1001/archinte.165.22.2618 16344419

[CIT0028] Hoogland, A. I., Mansfield, J., Lafranchise, E. A., Bulls, H. W., Johnstone, P. A., & Jim, H. S. L. (2020). Ehealth literacy in older adults with cancer. *Journal of Geriatric Oncology*, *11*(6), 1020–1022. doi:10.1016/j.jgo.2019.12.015 31917114 PMC8320530

[CIT0029] IBM Corp. (2015). *IBM SPSS Statistics for Windows, Version 23.0*.

[CIT0030] ICDC. (2015). *Israel National Health Interview Survey-3*. http://www.health.gov.il/PublicationsFiles/INHIS_3main_findings.pdf

[CIT0031] Idler, E. L., & Benyamini, Y. (1997). Self-rated health and mortality: A review of twenty-seven community studies. *Journal of Health and Social Behavior*, *38*(1), 21–37. doi:10.2307/2955359 9097506

[CIT0032] Israel Central Bureau of Statistics. (2020a). Computer *and Interent use of 20+ by demographic characteristics.* https://www.cbs.gov.il/he/publications/doclib/2020/17.shnatonsciencetechnologycommunication/st17_19x.pdf

[CIT0033] Israel Central Bureau of Statistics. (2020b). *The Muslim population in Israel.* Media release 230/2020 (Hebrew).

[CIT0034] Jones, R. (2013). Development of a questionnaire and cross-sectional survey of patient eHealth readiness and eHealth inequalities. *Medicine*, *2.0*(2(2)), e9. doi:10.2196/med20.2559 PMC4084763 25075244

[CIT0035] Karnoe, A., & Kayser, L. (2015). How is eHealth literacy measured and what do the measurements tell us? A systematic review. *Knowledge Management and E-Learning*, *7*(4), 576–600.

[CIT0036] Kim, K. A., Kim, Y. J., & Choi, M. (2018). Association of electronic health literacy with health-promoting behaviors in patients with type 2 diabetes: A cross-sectional study. *CIN - Computers Informatics Nursing*, *36*(9), 438–447. doi:10.1097/CIN.0000000000000438 29742548

[CIT0037] Knapp, C., Madden, V., Wang, H., Sloyer, P., & Shenkman, E. (2011). Internet use and eHealth literacy of low-income parents whose children have special health care needs. *Journal of Medical Internet Research*, *13*(3), 1–15. doi:10.2196/jmir.1697 PMC3222184 21960017

[CIT0038] Koopman, R. J., Petroski, G. F., Canfield, S. M., Stuppy, J. A., & Mehr, D. R. (2014). Development of the PRE-HIT instrument: Patient readiness to engage in health information technology. *BMC Family Practice*, *15*(18), doi:10.1186/1471-2296-15-18 PMC3916695 24472182

[CIT0039] Lev-On, A., & Lissitsa, S. (2010). *Digital divide, Israel 2008*. Mediterranean Conference on Information Systems 2010 (MCIS 2010), Paper 54, 1–15. http://aisel.aisnet.org/mcis2010/54/

[CIT0040] Levin-Zamir, D., Baron-Epel, O. B., Cohen, V., & Elhayany, A. (2016). The association of aealth literacy with health behavior, socioeconomic indicators, and self- assessed health from a national adult survey in Israel. *Journal of Health Communication*, *21*, 61–68. doi:10.1080/10810730.2016.1207115 27669363

[CIT0041] Lissitsa, S. (2015). Patterns of digital uses among Israeli Arabs – between citizenship in modern society and traditional cultural roots. *Asian Journal of Communication*, *25*(5), 447–464. doi:10.1080/01292986.2014.981555

[CIT0042] Lissitsa, S., & Chachashvili-Bolotin, S. (2014). Use of the Internet in capital enhancing ways – ethnic differences in Israel and the role of language proficiency. *International Journal of Internet Science*, *9*(1), 9–30.

[CIT0043] Lissitsa, S., & Chachashvili-Bolotin, S. (2019). The effect of digital variables on perceived employability in an ethnic minority and the hegemonic group. *Israel Affairs*, *25*(6), 1082–1104. doi:10.1080/13537121.2019.1670471

[CIT0044] Mackert, M., Champlin, S. E., Holton, A., Muñoz, I. I., Damásio, M. J., Munoz, I. I., … Damásio, M. J. (2014). Ehealth and health literacy: A Research methodology review. *Journal of Computer-Mediated Communication*, *19*(3), 516–528. doi:10.1111/jcc4.12044

[CIT0045] Maroney, K., Curtis, L. M., Opsasnick, L., Smith, K. D., Eifler, M. R., Moore, A., … Patzer, R. E. (2020). Ehealth literacy and web-based patient portal usage among kidney and liver transplant recipients. *Clinical Transplantation*, *2020*, 1–12. doi:10.1111/ctr.14184 33278846

[CIT0046] Mesch, G. S. (2012). Minority status and the use of computer-mediated communication: A test of the social diversification hypothesis. *Communication Research*, *39*(3), 317–337. doi:10.1177/0093650211398865

[CIT0047] Mesch, G. S. (2015). Ethnic origin and access to electronic health services. *Health Informatics Journal*, *22*(4), 791–803. doi:10.1177/1460458215590863 26261219

[CIT0048] Mesch, G. S., & Talmud, I. (2011). Ethnic differences in Internet access. *Information Communication Society*, *2015*, 1–27. doi:10.1080/1369118X.2011.562218

[CIT0049] Mitsutake, S., Shibata, A., Ishii, K., & Oka, K. (2016). Associations of eHealth literacy with health behavior among adult Internet users. *Journal of Medical Internet Research*, *18*(7), e192. doi:10.2196/jmir.5413 27432783 PMC4969548

[CIT0050] Mõttus, R., Johnson, W., Murray, C., Wolf, M. S., Starr, J. M., & Deary, I. J. (2014). Towards understanding the links between health literacy and physical health. *Health Psychology*, *33*(2), 164–173. doi:10.1037/a0031439 23437854

[CIT0051] Neter, E., & Brainin, E. (2012). Ehealth literacy: Extending the digital divide to the realm of health information. *Journal of Medical Internet Research*, *14*(1), e19. doi:10.2196/jmir.1619 22357448 PMC3374546

[CIT0052] Neter, E., & Brainin, E. (2017). Perceived and performed eHealth literacy: Survey and simulated performance test. *JMIR Human Factors*, *4*(1), e2. doi:10.2196/humanfactors.6523 28096068 PMC5285606

[CIT0053] Neter, E., & Brainin, E. (2019). Association between health literacy, eHealth literacy, and health outcomes among patients with long-term conditions: A systematic review. *European Psychologist*, *24*(1), doi:10.1027/1016-9040/a000350

[CIT0054] Neter, E., Brainin, E., & Baron-Epel, O. (2015). The dimensionality of health literacy and eHealth literacy. *Bulletin of the European Health Psychologist*, *17*(6), 275–280.

[CIT0055] Neter, E., Brainin, E., & Baron-Epel, O. (2018). The third digital divide in the health domain: Is Internet use for health purposes associated with health benefits? *Emerald Media Studies: E-Health: Current Evidence, Promises, Perils, and Future Directions*, *15*, 153–175. doi:10.1108/S2050-206020180000015011

[CIT0056] Nguyen, T. H., Paasche-Orlow, M. K., & McCormack, L. A. (2017). The state of the science of health literacy measurement. *Information Services & Use*, *37*(2), 189–203. doi:10.3233/ISU-170827 PMC6082165 28972507

[CIT0057] Norgaard, O., Furstrand, D., Klokker, L., Karnoe, A., Batterham, R., Kayser, L., & Osborne, R. H. (2015). The e-health literacy framework: A conceptual framework for characterizing e-health users and their interaction with e-health systems. *Knowledge Management & E-Learning*, *7*(4), 522–540.

[CIT0058] Norman, C. (2011). Ehealth literacy 2.0: Problems and opportunities with an evolving concept. *Journal of Medical Internet Research*, *13*(4), e125. http://www.jmir.org/2011/4/e125/?trendmd-shared=1 22193243 10.2196/jmir.2035 PMC3278111

[CIT0059] Norman, C. D., & Skinner, H. A. (2006). eHEALS: The eHealth literacy scale. *Journal of Medical Internet Research*, *8*(4), 1–11. doi:10.2196/jmir.8.4.e27 PMC1794004 17213046

[CIT0060] Nutbeam, D. (2000). Health literacy as a public health goal: A challenge for contemporary health education and communication strategies into the 21st century. *Health Promotion International*, *15*(3), 259–267. doi:10.1093/heapro/15.3.259

[CIT0061] Nutbeam, D. (2008). The evolving concept of health literacy. *Social Science & Medicine*, *67*(12), 2072–2078. doi:10.1016/j.socscimed.2008.09.050 18952344

[CIT0062] Paasche-Orlow, M. K., & Wolf, M. S. (2007). The causal pathways linking health literacy to health outcomes. *American Journal of Health Behavior*, *31*(1), S19–S26. doi:10.5993/AJHB.31.s1.4 17931132

[CIT0063] Parker, R. M, Baker, D.W, Williams, M.V, & Nurss, J.R. (1995). The test of functional health literacy in adults. Journal of general internal medicine, *10*(10), 537–541. 8576769 10.1007/BF02640361

[CIT0064] Parker, R. M., Ratzan, S. C., & Lurie, N. (2003). Health literacy: A policy challenge for advancing high-quality health care. *Health Affairs*, *22*(4), 147–153. doi:10.1377/hlthaff.22.4.147 12889762

[CIT0065] Rudd, R., Kirsch, I. S., & Yamamoto, K. (2004). Literacy and health in America. Policy information report. In *Educational Testing Service*. http://www.ets.org/Media/Research/pdf/PICHEATH.pdf

[CIT0066] Schillinger, D. (2002). Association of health literacy with diabetes outcomes. *JAMA*, *288*(4), 475–478. doi:10.1001/jama.288.4.475 12132978

[CIT0067] Shiferaw, K. B., Tilahun, B. C., Endehabtu, B. F., Gullslett, M. K., & Mengiste, S. A. (2020). E-health literacy and associated factors among chronic patients in a low-income country: A cross-sectional survey. *BMC Medical Informatics and Decision Making*, *20*(1), 1–9. doi:10.1186/s12911-020-01202-1 32762745 PMC7407428

[CIT0068] Smooha, S. (1997). Ethnic democracy: Israel as an archetype. *Israel Studies*, *2*(2), 198–241. doi:10.2979/ISR.1997.2.2.198

[CIT0069] Sørensen, K, den Broucke, S, Pelikan, J.M, Fullam, J, Doyle, G, Slonska, Z, … Brand, H. (2013). Measuring health literacy in populations: illuminating the design and development process of the European Health Literacy Survey Questionnair (HLS-EU-Q). BMC Public Health, *13*(1), 948. 24112855 10.1186/1471-2458-13-948 PMC4016258

[CIT0070] Sørensen, K., den Broucke, S., Pelikan, J. M., Fullam, J., Doyle, G., Slonska, Z., … Brand, H. (2013). Measuring health literacy in populations: Illuminating the design and development process of the European Health literacy survey questionnair (HLS-EU-Q). *BMC Public Health*, *13*(1), 948. 24112855 10.1186/1471-2458-13-948 PMC4016258

[CIT0071] Stellefson, M., Paige, S. R., Alber, J. M., Chaney, B. H., Chaney, D., Apperson, A., & Mohan, A. (2019). Association between health literacy, electronic health literacy, disease-specific knowledge, and health-related quality of life among adults with chronic obstructive pulmonary disease: Cross-sectional study. *Journal of Medical Internet Research*, *21*(6), doi:10.2196/12165 PMC6592488 31172962

[CIT0072] Tennant, B., Stellefson, M., Dodd, V., Chaney, B., Chaney, D., Paige, S., & Alber, J. (2015). Ehealth literacy and Web 2.0 health information seeking behaviors among baby boomers and older adults. *Journal of Medical Internet Research*, *17*(3), e70. doi:10.2196/jmir.3992 25783036 PMC4381816

[CIT0073] Van Den Broucke, S., Levin-Zamir, D., Schaeffer, D., Pettersen, K., Guttersrud, Ø, Finbråten, H., … Pelikan, J. (2020). Digital health literacy in general populations – An international comparison. *European Journal of Public Health*, *30*(Supplement 5), doi:10.1093/eurpub/ckaa165.124

[CIT0074] Van der Vaart, R., & Drossaert, C. H. C. C. (2017). Development of the digital health Literacy Instrument; measuring a broad spectrum of health 1.0 and health 2.0 skills. *JMIR*, *19*(1), 1–13. doi:10.2196/jmir.6709 PMC5358017 28119275

[CIT0075] Van Deursen, A., & Helsper, E. J. (2015). The third-level digital divide: Who benefits most from being online? *Communication and Information Technologies Annual*, *10*, 29–52. doi:10.1108/S2050-206020150000010002

[CIT0076] Wångdahl, J., Lytsy, P., Mårtensson, L., & Westerling, R. (2015). Health literacy and refugees’ experiences of the health examination for asylum seekers - A Swedish cross-sectional study health behavior, health promotion and society. *BMC Public Health*, *15*(1), 1. doi:10.1186/s12889-015-2513-8 26596793 PMC4657287

[CIT0077] Weiss, B.D, Mays, M.Z, Martz, W, Castro, K.M, DeWalt, D.A, Pignone, M.P, & Hale, F.A. (2005). Quick assessment of literacy in primary care: the newest vital sign. The Annals of Family Medicine, *3*(6), 514–522. 16338915 10.1370/afm.405 PMC1466931

[CIT0078] Williams, M. V., Baker, D. W., Honig, E. G., Lee, T. M., & Nowlan, A. (1998). Inadequate literacy is a barrier to asthma knowledge and self-care. *Chest*, *114*(4), 1008–1015. doi:10.1378/chest.114.4.1008 9792569

[CIT0079] Woods, S. P., & Sullivan, K. L. (2019). Lower neurocognitive functioning disrupts the effective dse of internet-based health resources in HIV disease: The mediating effects of general health literacy capacity. *AIDS and Behavior*, *23*(3), 676–683. doi:10.1007/s10461-018-2350-8 30506473 PMC6408228

[CIT0080] World Health Organization. (1998). *Health Promotion*. World Health Organization Geneva.

[CIT0081] Yin, H. S., Dreyer, B. P., Foltin, G., van Schaick, L., & Mendelsohn, A. L. (2007). Association of low caregiver health literacy with reported use of nonstandardized dosing instruments and lack of knowledge of weight-based dosing. *Ambulatory Pediatrics*, *7*(4), 292–298. doi:10.1016/j.ambp.2007.04.004 17660100

[CIT0082] Zamora, H., & Clingerman, E. M. (2011). Health literacy among older adults: A systematic literature review. *Journal of Gerontological Nursing*, *37*(10), 41–51. doi:10.1093/her/cys067 21634314

[CIT0083] Zikmund-Fisher, B. J., Exe, N. L., & Witteman, H. O. (2014). Numeracy and literacy independently predict patients’ ability to identify out-of-range test results. *Journal of Medical Internet Research*, *16*(8), e187. doi:10.2196/jmir.3241 25135688 PMC4137189

